# Enhanced Hyperthermia Induced by MDMA in Parkin Knockout Mice

**DOI:** 10.2174/157015911795016985

**Published:** 2011-03

**Authors:** Y Takamatsu, H Shiotsuki, S Kasai, S Sato, T Iwamura, N Hattori, K Ikeda

**Affiliations:** 1 Division of Psychobiology, Tokyo Institute of Psychiatry, 2-1-8 Kamikitazawa, Setagaya-ku, Tokyo 156-8585, Japan; 2 Department of Neurology, Juntendo University School of Medicine, 2-1-1 Hongo, Bunkyou-ku, Tokyo 113-8421, Japan; 3 Matsuyama University College of Pharmaceutical Sciences, 4-2 Bunkyo-cho, Matsuyama, Ehime 790-8578, Japan

**Keywords:** Hyperthermia, knockout, mice, MDMA, parkin.

## Abstract

MDMA (3,4-methylenedioxymethamphetamine) is reportedly severely toxic to both dopamine (DA) and serotonin neurons. MDMA significantly reduces the number of DA neurons in the substantia nigra, but not in the nucleus accumbens, indicating that MDMA causes selective destruction of DA neurons in the nigrostriatal pathway, sparing the mesolimbic pathway. Parkinson’s disease (PD) is a neurodegenerative disorder of multifactorial origin. The pathological hallmark of PD is the degeneration of DA neurons in the nigrostriatal pathway. Mutations in the parkin gene are frequently observed in autosomal recessive parkinsonism in humans. Parkin is hypothesized to protect against neurotoxic insult, and we attempted to clarify the role of parkin in MDMA-induced hyperthermia, one of the causal factors of neuronal damage, using parkin knockout mice. Body temperature was measured rectally before and 15, 30, 45, and 60 min after intraperitoneal injection of MDMA (30 mg/kg) at an ambient temperature of 22 ± 2°C. Significantly enhanced hyper-thermia after MDMA injection was observed in heterozygous and homozygous parkin knockout mice compared with wildtype mice, suggesting that parkin plays a protective role in MDMA neurotoxicity.

## INTRODUCTION

The amphetamine derivative 3,4-methylenedioxymet-hamphetamine (MDMA) is abused by young adults despite its potentially neurotoxic effects and psychiatric complications. MDMA produces a rapid enhancement of serotonin and dopamine (DA) release in the brain [1, 2]. Administration of MDMA in mice is well known to produce acute hyperthermia and degeneration of striatal DA nerve terminals [3]. Recently, Granado and colleagues [4] reported that MDMA produces a significant decrease in the number of tyrosine hydroxylase (TH)-immunoreactive neurons in the substantia nigra. This decrease was accompanied by a dose-dependent decrease in TH- and DA transporter (DAT)-immunoreactivity in the striatum. MDMA significantly reduces TH- and DAT-immunoreactivity in the striatum, but not in the nucleus accumbens, indicating that MDMA causes selective destruction of DA neurons in the nigrostriatal pathway, sparing the mesolimbic pathway. The degree of long-term neurodegeneration produced by MDMA appears to be closely related to the magnitude of the hyperthermic response [5]. Attenuation of the hyperthermia alleviates MDMA-induced loss of striatal dopamine [3].

Parkinson’s disease (PD) is the most common neurodegenerative movement disorder. The major pathological hallmark of PD is the degeneration of DAergic neurons in the substantia nigra that innervate the striatum. The major symptoms of PD include tremor, bradykinesia, cogwheel rigidity, and postural instability, which arise from the degeneration of DAergic neurons in the substantia nigra. PD is a neurodegenerative disorder of multifactorial origin, and mutations in the gene encoding parkin, an E3 ubiquitin-protein ligase [6], are frequently observed in autosomal recessive parkinsonism in humans. The loss of parkin function has been suggested to result in aberrant accumulation of parkin substrate proteins [6]. Accumulation of these proteins has been postulated to confer toxicity to DAergic neurons in the substantia nigra [7].

In the present study, we hypothesized that parkin protects against neurotoxic insult, and we attempted to clarify the role of parkin in MDMA-induced hyperthermia, one of the causal factors of neuronal damage, using parkin knockout mice.

## MATERIALS AND METHODS

### Mice

Wildtype, heterozygous, and homozygous parkin knockout mice were prepared from heterozygous/heterozygous parkin knockout mouse crosses (21-37 g, 12-29 weeks of age). Mice were housed in an animal facility maintained at 22 ± 2ºC and 55 ± 5% relative humidity under a 12/12 h light/dark cycle with lights on at 8:00 a.m. Food and water were available *ad libitum*. All behavioral testing was conducted during the light cycle. The experimental procedures and housing conditions were approved by the Institutional Animal Care and Use Committee of the Tokyo Institute of Psychiatry, and all animals were treated humanely in accordance with our institutional animal experimentation guidelines.

### Body Temperature Measurement

Rectal temperature measurement was performed using a digital thermometer (BAT-12; Physitemp Instruments Inc., Clifton, NJ, USA) with 0.1ºC accuracy and a rectal probe for mice (RET-3, Physitemp Instrument Inc.). Each mouse was lightly restrained by hand for approximately 20 s while the probe was inserted approximately 2 cm into the rectum and a steady reading was obtained. Body temperature was measured rectally before and 15, 30, 45, and 60 min after intraperitoneal (i.p.) injection of MDMA (30 mg/kg) at an ambient temperature of 22 ± 2ºC.

### Drugs

MDMA was synthesized at Matsuyama University College of Pharmaceutical Sciences and freshly dissolved in saline. MDMA and vehicle were administered in a volume of 0.1 ml/10 g body weight.

### Statistical Analysis

Mean and standard error were calculated from the values of 12-17 subjects. Changes in body temperature were analyzed by repeated-measures analysis of variance (ANOVA) followed by Scheffe’s *post hoc* test. Baseline temperature and changes in body temperature areas-under-the-curve (AUC) were analyzed by one-way ANOVA and Scheffe’s *post hoc *test.

## RESULTS

### Baseline Body Temperature in Parkin Knockout Mice

**Fig. (1):**
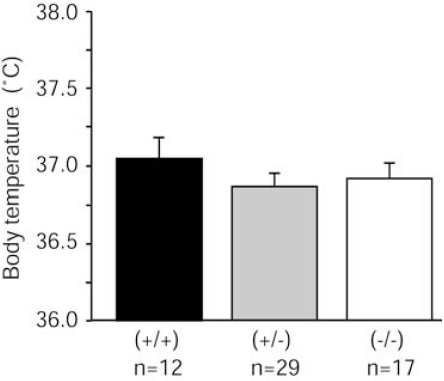
No significant differences in baseline body temperature were observed among wildtype, heterozygous, and homozygous parkin knockout mice. Body temperature prior to MDMA injection did not significantly differ among genotypes. Baseline body temperature was analyzed by one-way ANOVA (*F*_2,55_ = 0.629, *p* = 0.5369) at an ambient temperature of 22 ± 2°C.

Baseline body temperature was measured before MDMA injection at room temperature (22 ± 2ºC). No significant difference in baseline body temperature was observed among wildtype, heterozygous, and homozygous parkin knockout mice (Fig. **1**).

### No Sex Differences in MDMA-Induced Hyperthermia

**Fig. (2):**
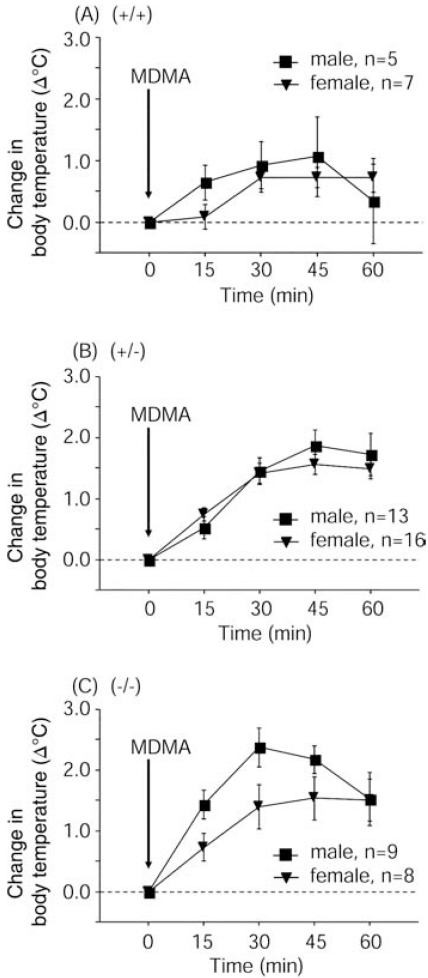
Similar MDMA-induced (30 mg/kg, i.p.) hyperthermia was observed in male and female mice within each genotype. Body temperature areas-under-the-curve were analyzed by repeated-measures ANOVA. (**A**) Sex, *F*_1,10_ = 0.181, *p* = 0.6796; Time, *F*_4,40_ = 4.741, *p* = 0.0032; Sex × Time interaction, *F*_4,40_ = 1.124, *p* = 0.3587. (**B)** Sex, *F*_1,27_ = 0.134, *p* = 0.7170; Time, *F*_4,108_ = 62.705, *p* < 0.0001; Sex × Time interaction, *F*_4,108_ = 1.231, *p* = 0.3021. (**C**) Sex, *F*_1,15_ = 2.350, *p* = 0.1461; Time, F_4,60_ = 26.22, *p* < 0.0001; Sex × Time interaction, *F*_4,60_ = 2.059, *p* = 0.0974.

Body temperature was measured 15, 30, 45, and 60 min after i.p. injection of MDMA (30 mg/kg) at an ambient temperature of 22 ± 2ºC. No significant differences in MDMA-induced hyperthermia were observed between males and females within each genotype (Fig. **2**).

### Enhancement of MDMA-Induced Hyperthermia in Parkin Knockout and Heterozygous Mice

**Fig. (3):**
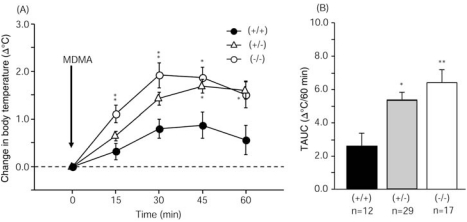
Enhanced MDMA-induced hyperthermia in heterozygous and homozygous parkin knockout mice compared with wildtype mice. (**A**) Change in body temperature in mice injected with MDMA (30 mg/kg, i.p.). Body temperature areas-under-the-curve were analyzed by repeated-measures ANOVA (Genotype, *F*_2,55_ = 6.746, *p* = 0.0024; Time, *F*_4,220_ = 61.267, *p* < 0.0001; Genotype × Time interaction, *F*_8,220_ = 3.664, *p* = 0.0005) followed by Scheffe’s *post hoc* test (*p < 0.05, **p < 0.01). (**B**) Change in body temperature areas-under-the-curve (TAUC) shown as an integration of the temperature *vs*. time curve shown in panel A. TAUC values were analyzed by one-way ANOVA (*F*_2,55_ = 6.746, *p* = 0.0024) followed by Scheffe’s *post hoc* test (*p < 0.05, **p < 0.01).

Body temperature gradually increased from baseline after MDMA injection in all genotype groups. MDMA significantly enhanced hyperthermia from 15 to 45 min after injection in parkin knockout mice and from 45 to 60 min after injection in heterozygous mice compared with wildtype mice (Fig. **3A**). MDMA produced hyperthermia, with a maximum increase of 0.9ºC (37.9ºC) 45 min after injection in wildtype mice, 1.7ºC (38.6ºC) 45 min after injection in heterozygous mice, and 1.9ºC (38.8ºC) 30 min after injection in parkin knockout mice. Body temperature AUC values reflected significantly enhanced hyperthermia in parkin knockout and heterozygous mice compared with wildtype mice (Fig. **3B**).

## DISCUSSION

In the present study, significantly enhanced MDMA-induced hyperthermia was observed in parkin knockout and heterozygous mice compared with wildtype mice (Fig. **3B**). The enhanced MDMA-induced hyperthermia in parkin knockout mice supports the hypothesis that parkin protects against MDMA-induced neurotoxic insult.

Hyperthermia is one of the major symptoms of acute MDMA-induced toxicity, which has been shown to be affected by body temperature [3]. MDMA produces a rapid enhancement of DA release in the striatum [1] and preoptic anterior hypothalamus [8]. MDMA-induced hyperthermia was blocked by a DA D_1_ receptor antagonist [9]. Moreover, both the hyperthermia and augmented DA levels in the preoptic anterior hypothalamus after i.p. MDMA injection were significantly reduced by pretreatment with a D_1_ antagonist [8]. Interestingly, Sato *et al.* (2006) [10] reported that D_1_ receptor levels in the striatum in parkin knockout mice was higher than in wildtype mice, although no change in TH-positive substantia nigra neurons was found in parkin knockout mice, and no significant decrease in DAT levels was observed in the striatum. Therefore, the enhanced MDMA-induced hyperthermia observed in the present study may be attributable to increased levels of D_1_ receptors in parkin knockout mice.

Sato *et al.* (2006) [10] also suggested that presynaptic neurons (i.e., DAergic neurons) are functionally impaired in parkin knockout mice. DA synthesis is significantly decreased and methamphetamine-induced DA release is reduced in parkin knockout mice. Considering that DAergic neurons in the substantia nigra are severely damaged in PD patients, the enhanced MDMA-induced hyperthermia in parkin knockout mice may be attributable to functional impairment of DAergic neurons, although the relationship between hyperthermia and DAergic neuron dysfunction remains to be elucidated.

Additionally, we found no significant difference in baseline body temperature among wildtype, heterozygous, and homozygous parkin knockout mice (Fig. **1**). These data suggest that parkin does not play a crucial role in the system that maintains basal body temperature.

In conclusion, MDMA-induced hyperthermia was enhanced in parkin knockout and heterozygous mice compared with wildtype mice. Parkin is hypothesized to be critical for protecting DAergic neurons from toxic insult, and the present results suggest that parkin plays a protective role against MDMA-induced DAergic neuron neurotoxicity.

## ABBREVIATIONS

ANOVA: = Analysis of variance
DA: = Dopamine
DAT: = Dopamine transporter
MDMA: = 3,4-methylenedioxymethamphetamine
PD: = Parkinson’s disease
TH: = Tyrosine hydroxylase
